# Multiple Extensive Brachial Plexus Schwannomas

**DOI:** 10.7759/cureus.101006

**Published:** 2026-01-07

**Authors:** Arshi Kaur, Maunil Mullick, Raja N Jani, Ramin Hamidi, Mercia J Bezerra Gondim, Brian J Williams

**Affiliations:** 1 Neurological Surgery, University of Louisville School of Medicine, Louisville, USA; 2 Neurological Surgery, University of Louisville, Louisville, USA; 3 Diagnostic Radiology, University of Louisville, Louisville, USA; 4 Pathology and Laboratory Medicine, University of Louisville, Louisville, USA

**Keywords:** brachial plexus, multifocal brachial plexus schwannoma, peripheral schwannoma, rare, schwannoma

## Abstract

Schwannomas are typically slow-growing, benign neoplasms with peak incidence between the third and sixth decades of life. While commonly located in the head, neck, and flexor surfaces, schwannomas of the brachial plexus are rare. We present a unique case of a multifocal brachial plexus schwannoma in a 41-year-old male. A supraclavicular and infraclavicular surgical approach revealed four distinct masses that displaced rather than invaded the brachial plexus. Postoperatively, the patient experienced partial motor and sensory deficits, with gradual improvement. A routine follow-up MRI three months following the resection revealed residual schwannomas left in situ due to positive intraoperative motor response. This case highlights the unique presentation and challenges of multifocal brachial plexus schwannomas.

## Introduction

Schwannomas typically align with World Health Organization (WHO) Grade I tumors, distinguished by a slow-growing, benign nature and a well-circumscribed, encapsulated structure formed from differentiated Schwann cells within the nerve sheath [1-5]. The mass appears homogeneous, with a yellow or tan color, and grows on the surface of the parent nerve, displacing it eccentrically [1,6,7]. Schwannomas exhibit peak incidence during the third to sixth decades of life, occurring with equal frequency in both sexes, except for intracranial lesions, noted as being twice as prevalent in females compared to males [6-9]. Clinical manifestations are influenced by tumor location, geometry, and growth rate [6]. Symptoms primarily arise from the compression of adjacent structures, particularly the nerve from which the tumor originates [9]. In all, 90% of schwannomas occur as solitary, sporadic cases [1,6,8,9]. The remaining are linked to familial cancer syndromes, notably (i) schwannomatosis, defined by germline variants in SMARCB1 or LZTR1, both located on chromosome 22q11; (ii) neurofibromatosis type 1 (NF1), resulting from germline variants on NF1 on chromosome 17q11, affecting the function of the cytoplasmic protein, neurofibromin; and (iii) neurofibromatosis type 2 (NF2), an autosomal dominant disorder driven by mutations in the NF2 tumor suppressor gene on chromosome 22, leading to a deficiency of the merlin protein [1,9-11]. Beyond these neoplastic syndromes, the etiology of schwannomas remains largely unclear [6]. Possible but undetermined risk factors include (i) a family history of spinal cancer linked to the development of spinal schwannomas, suggesting a potential genetic predisposition, and (ii) radiation exposure proposed as a contributing factor [6,8]. Schwannomas can affect both the central and peripheral nervous systems and are most commonly located in the head, neck, and flexor surfaces of the extremities [3,4]. As extracranial tumors, schwannomas are the most prevalent form of peripheral nerve lesions; however, the involvement of the brachial plexus is uncommon, accounting for approximately 5% of all schwannoma cases [1,2].

Here, we present a rare case of a 41-year-old male with multifocal brachial plexus schwannomas.

## Case presentation

**Figure 1:**
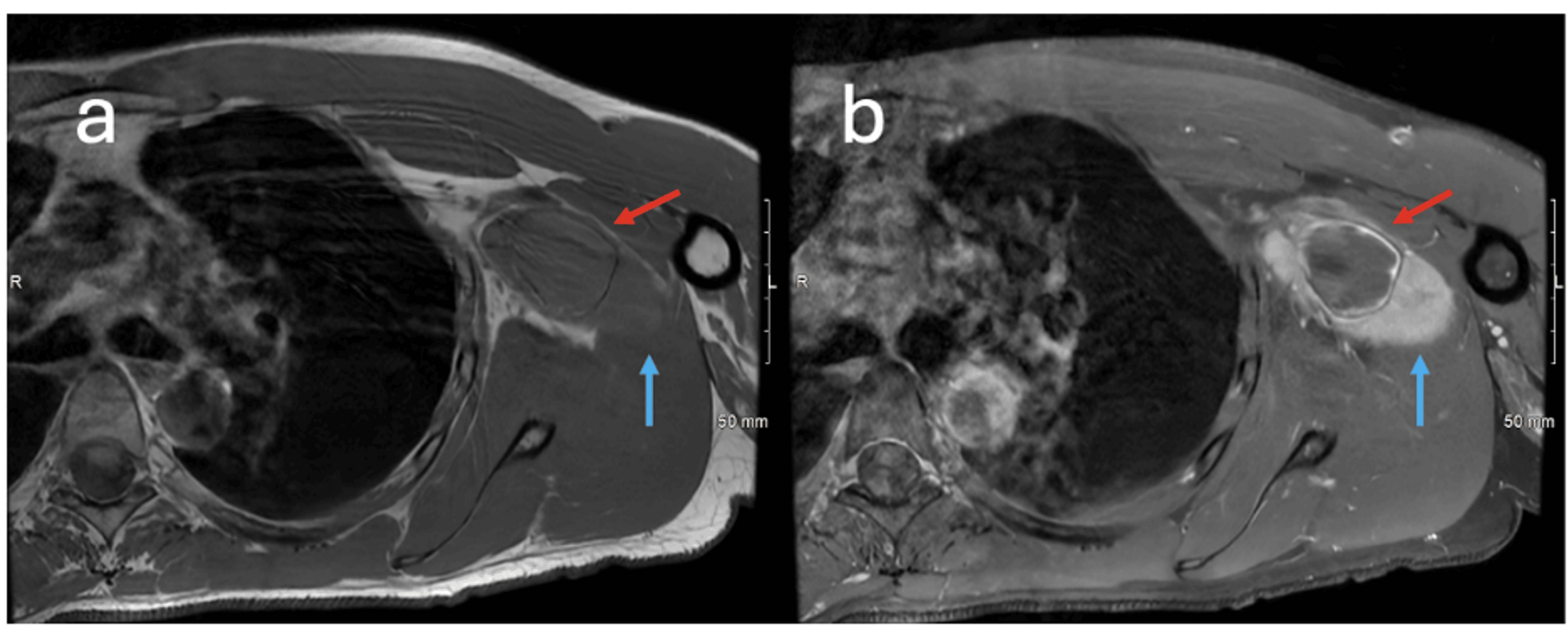
Axial T1 and T1 fat-saturated post-contrast MRI of the left brachial plexus demonstrating cystic and solid lesions Axial T1 image of the left brachial plexus (a) demonstrates a complex T1 dark cystic (red arrow) and a T1 isointense to muscle (blue arrow) lesion in the region of the left brachial plexus. Axial T1 fat-saturated post-contrast image of the same level (b) demonstrates faint peripheral enhancement of the cystic lesion (red arrow) and solid enhancement of the adjacent solid lesion (blue arrow).

**Figure 2:**
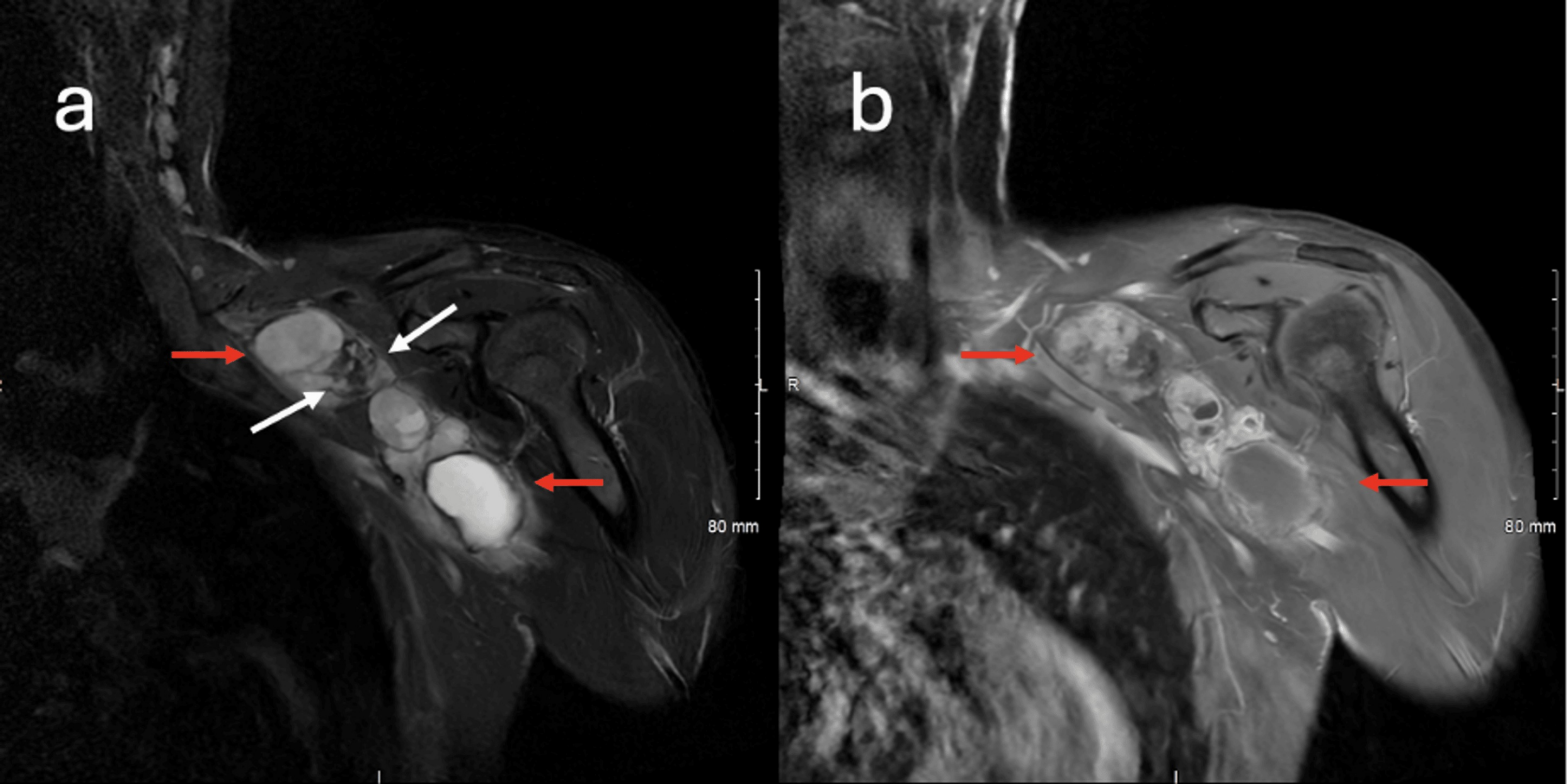
Coronal T2 STIR and post-contrast T1 fat-saturated MRI of the left brachial plexus demonstrating a heterogeneous lesion and variable cyst enhancement Coronal T2 STIR image of the left brachial plexus (a) demonstrates the complex heterogeneous lesion (red arrows) with blood products (white arrows), presumed secondary to hemosiderin deposition. Coronal T1 fat-saturated post-contrast image at the same level (b) demonstrates areas of enhancement within the lesion. Note that the more cephalad cyst (upper red arrow) demonstrates enhancement, while another area of cystic change (lower red arrow) demonstrates minimal enhancement.

**Figure 3:**
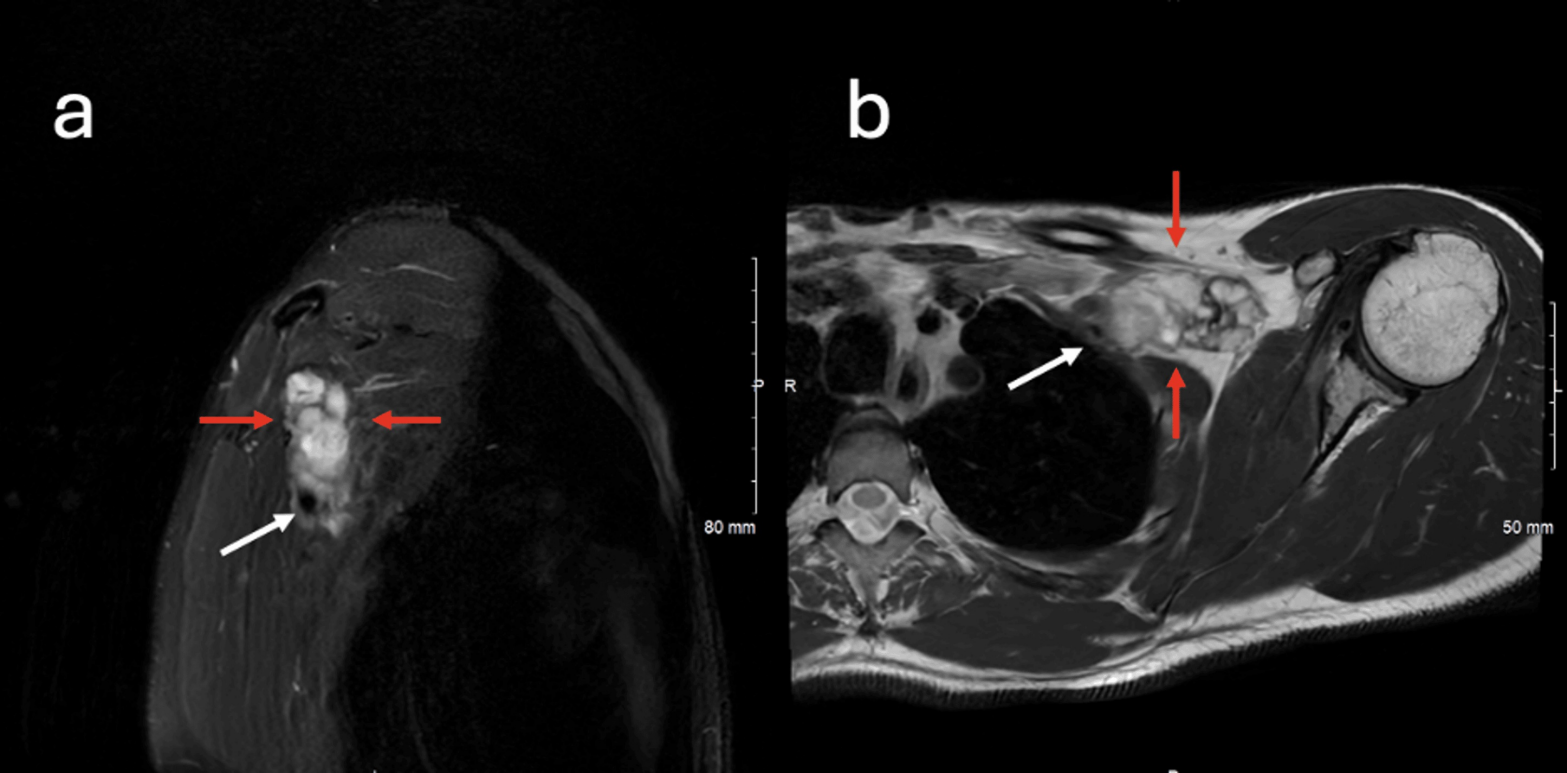
Sagittal and axial T2 MRI of the left brachial plexus demonstrating tumor relationship to axillary and brachial arteries Sagittal T2 fat-saturated image of the left brachial plexus (a) demonstrates the relationship of the left axillary artery (white arrow) with the adjacent tumor (red arrow). Axial T2 image of the left brachial plexus (b) demonstrates the relationship between the left brachial artery (white arrow) and the cystic tumor (red arrows). Note that for purposes of surgical planning, the lesion is cephalad and anterior to the left brachial artery.

A 41-year-old male presented at the University of Louisville Hospital, Louisville, KY, in April 2024 with a gradually enlarging left axillary mass limiting his daily activities. The patient reported that he underwent a biopsy at an outside facility a few years prior and was told the lesion was benign; however, records from this encounter were inaccessible. At the current presentation, the patient reported neuropathic pain along his whole arm with occasional numbness and tingling. He did not present with obvious weakness and had no significant family history of neurofibromatosis. Preoperative physical exam revealed a firm palpable egg-sized mass with limited mobility in the left axilla and with no supraclavicular adenopathy. Neurological testing revealed normal sensory and motor function of the bilateral upper extremities. MRI in April of 2024 demonstrated a heterogeneous mixed solid and cystic mass measuring 3.4 cm centered in the region of the left brachial plexus. Blood products consistent with hemosiderin, suggesting chronic hemorrhage, were present in the lesion, and the pattern of enhancement was also heterogeneous, with varying degrees of enhancement in cystic and solid portions. The tumor was cephalad and anterior to, as well as approximating, the axillary artery and vein. The presence of T1 isointense and cystic components with heterogeneous contrast enhancement suggested a schwannoma with differentials including inflammatory and infectious neoplastic processes such as lymphoma or metastasis (Figures 1-3). Subsequent ultrasound-guided soft tissue biopsy of the left axillary mass in June 2024 at the University of Louisville Hospital was consistent with a schwannoma. The tumor was surgically resected in September of 2024.

Surgical intervention

A supraclavicular and infraclavicular approach for resection of the left multifocal brachial plexus nerve sheath tumor was performed with the use of motor stimulation. The C5, C6, and C7 nerve roots were identified and followed to the clavicle. An infraclavicular approach to the brachial plexus was performed, and the brachial plexus cords, axillary vein, and artery were identified. Five lesions were identified; however, a non-stimulating window could not be identified in the last lesion, and resection was therefore terminated. During surgical exploration, the first large axillary lesion was identified. Intraoperative stimulation of the tumor capsule did not elicit a motor response, and the tumor was removed largely in one piece. Initial stimulation of the tumor capsule of the next proximal tumor elicited a motor response. A non-stimulating region of the capsule was identified, and dissection was performed in a deeper capsular plane with continuous stimulation. No further significant motor responses were observed, and the tumor was removed largely in one piece. Stimulation of the capsule of the next proximal tumor resulted in a motor response. A non-stimulating region of the capsule was identified, and dissection was performed in a deeper layer of the capsule with continuous stimulation. Stimulation was present along the dissection plane, and an intralesional technique was elected. No significant stimulation was appreciated within the tumor itself, and resection was terminated once gross disease was not evident through the window into the tumor capsule. Initial stimulation of the tumor capsule of the next proximal tumor elicited a motor response. A non-stimulating region of the capsule was identified, and dissection was performed in a deeper capsular plane with continuous stimulation. No further significant motor responses were observed, and the tumor was removed largely in one piece. As surgery proceeded superiorly, the clavicle began to limit the freedom of dissection. The next proximal tumor was identified, and a window that did not have significant motor stimulation was not appreciated. Therefore, the decision was made to terminate further resection.

Grossly, the four masses were noted to be firm, well-circumscribed, encapsulated pink-tan and yellow masses with cystic components that were eccentric in location to the native nerves as the tumors were dissected from the secondary nerve tissue. Masses were noted to displace the nerve rather than invade it. 

Histopathological examination 

**Figure 4:**
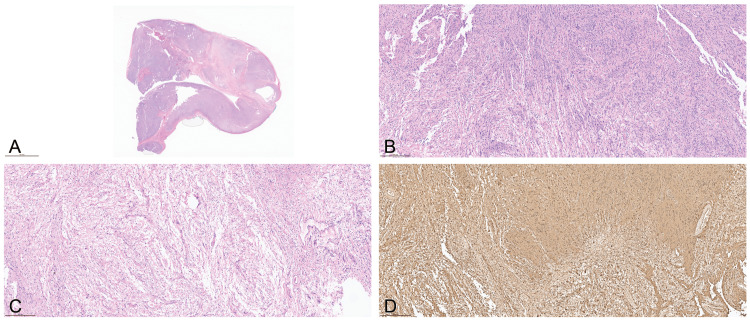
Histopathologic features of schwannoma with Antoni A and B areas and diffuse S100 positivity Histopathological features of the tumor. Hematoxylin and eosin (H&E)-stained section (A) demonstrates an encapsulated, well-circumscribed mass with Antoni A and Antoni B areas. The Antoni A regions (B) are cellular and show nuclear palisading, forming Verocay bodies, while Antoni B regions (B,C) represent the hypocellular component. Immunohistochemistry reveals diffuse positivity for S100 protein (D), further supporting the diagnosis.

Hematoxylin and eosin-stained sections of the tumor sample revealed an encapsulated, well-circumscribed mass with a zonal pattern of Antoni A and B areas (Figure 4A). The Antoni A areas were cellular, displaying nuclear palisading (Verocay bodies) (Figure 4B), in contrast to the Antoni B regions, which represented the hypocellular component (Figures 4B, 4C). Diffuse positive S100 staining by immunohistochemistry (Figure 4D) further supported the diagnosis.

Postoperative course and treatment

**Figure 5:**
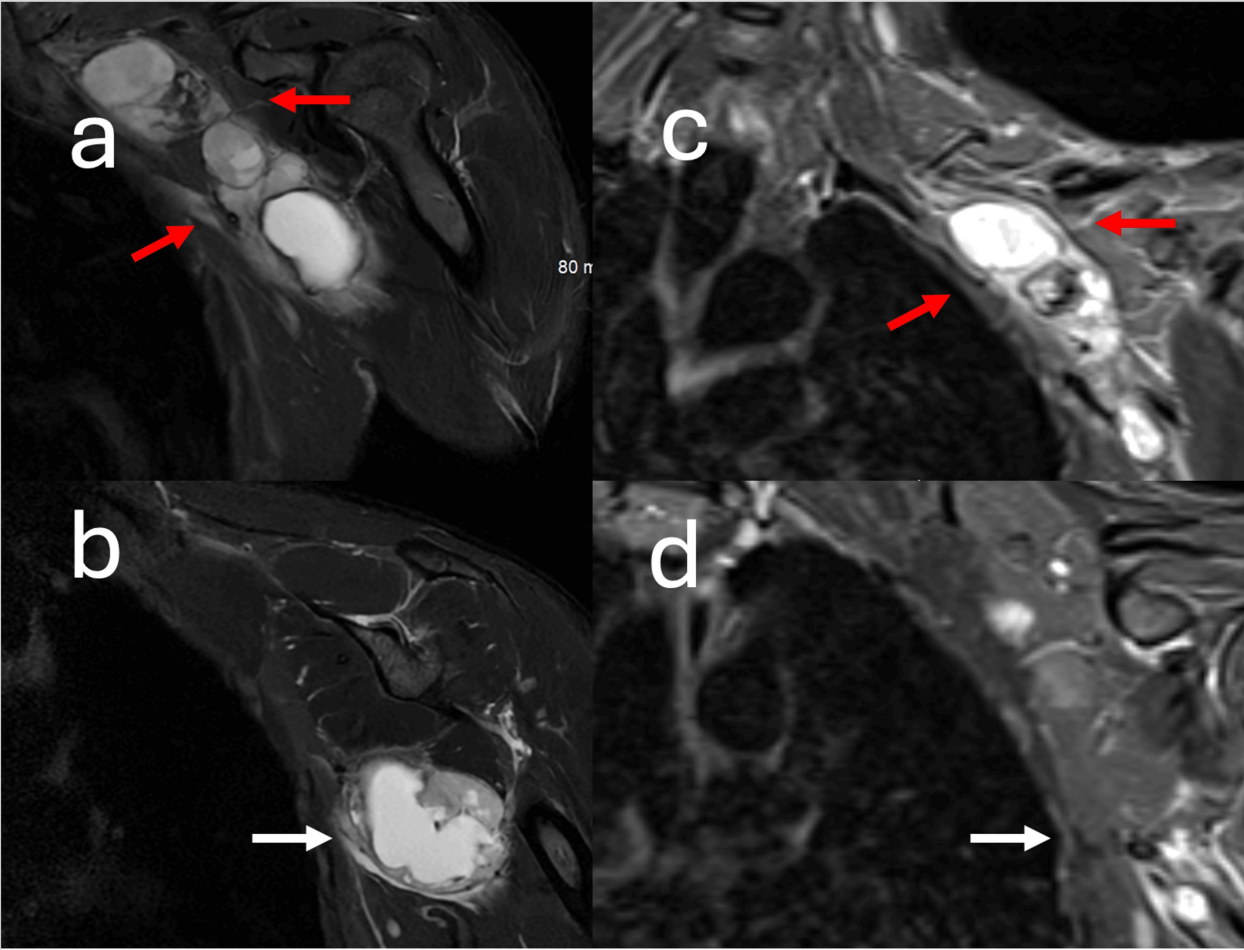
Coronal T2 fat-saturated images of the brachial plexus obtained pre-operatively and post-operatively Coronal T2 fat-saturated image of the brachial plexus obtained pre-operatively (a,b) and coronal T2 fat-saturated image of the brachial plexus obtained post-operatively. The surgical resection of schwannomas was performed with direct neuromonitoring, and only the schwannomas that had no motor function were removed. In (a), the red arrows point to a schwannoma that demonstrated nerve conduction following stimulation of the tumor capsule. The red arrows point to the same tumor in the post-operative exam (c). More anteriorly and caudally, stimulation of the tumor capsule marked with a white arrow in (b) demonstrated no nerve conduction. This lesion was removed entirely, with the surgical cavity manifest by the white arrow in (d). Each schwannoma that was resected was removed in its entirety, an expected finding as schwannomas typically wrap around a single nerve root.

Figure 5 illustrates pre-operative and post-operative coronal T2-weighted fat-saturated images of the brachial plexus.

Postoperatively, the patient noted mild left triceps weakness graded as four out of five. Overall, his neurological recovery was consistent with expected postoperative progression. At the three-month follow-up, MRI demonstrated a lobulated, enhancing, and heterogeneously T2-hyperintense mass extending along the brachial plexus from the mid-clavicular region to the axilla, consistent with a persistent nerve sheath tumor.

## Discussion

This case of multiple extensive brachial plexus schwannomas is a rare and complex presentation of a peripheral nerve sheath tumor. Schwannomas, typically benign and slow-growing, account for only 5% of all schwannoma cases when located in the brachial plexus, making this case particularly noteworthy [1,2]. The patient’s progressive symptoms, including pain, mass enlargement, and nerve compression, highlight the clinical challenge of diagnosing and managing schwannomas in anatomically intricate regions. The rarity of multifocal involvement, combined with the tumor’s close proximity to critical neurovascular structures, made this case both diagnostically and surgically complex.

Preoperative MRI was crucial in identifying the tumor’s complex characteristics and its relationship with adjacent structures. The presence of T1 isointense and cystic components with heterogeneous contrast enhancement suggested a schwannoma with possible degenerative or cystic changes [12]. Notably, the peripheral enhancement of the cystic lesion with adjacent solid enhancement reflects the typical imaging patterns of schwannomas, aiding in accurate preoperative diagnosis [12]. The relationship between the tumor and the brachial artery, demonstrated on axial T2 and sagittal T2 fat-saturated images, was essential for precise surgical planning, reducing the risk of vascular injury.

The surgical resection employed both a supraclavicular and infraclavicular approach, which was necessary given the multifocal and extensive nature of the tumor. The use of peripheral nerve stimulation was essential for preserving motor function, especially given the tumor’s proximity to the brachial plexus. Intraoperative findings of firm, well-circumscribed masses displacing but not invading the native nerve tissue reflect the characteristic behavior of schwannomas, which typically displace rather than infiltrate the nerve [13]. The successful separation of the tumors from the secondary nerve tissue is a testament to the precision and expertise required in peripheral nerve surgery.

Histopathological analysis confirmed the diagnosis, revealing a well-encapsulated, biphasic tumor with Antoni A and Antoni B areas. The Antoni A regions, characterized by nuclear palisading and Verocay bodies, are pathognomonic for schwannomas, while the Antoni B regions demonstrated hypocellularity and loose matrix tissue [14]. The diffuse S100 immunostaining further confirmed the Schwann cell origin of the tumor, aligning with the classical histopathological features of schwannomas [14].

Following surgical intervention, the patient’s postoperative course was notable for mild left triceps weakness (4/5) and diffuse sensory diminution in the left upper extremity relative to the contralateral side. The patient’s preoperative pain, which has been one of the indications for surgery, was significantly improved, likely due to the relief of pressure on the adjacent nervous system structures. These deficits were consistent with transient postoperative neuropraxia, likely reflecting intraoperative manipulation of involved nerve fibers during tumor resections. Gradual improvement in sensory function to approximately 90% of baseline suggests partial recovery of neural conduction and supports the expectation of continued improvement over time.

At three months postoperatively, MRI revealed a lobulated, enhancing, heterogeneously T2-hyperintense mass extending along the brachial plexus from the mid-clavicular region to the axilla, consistent with residual or recurrent nerve sheath tumor. The persistence of enhancing tissue along the plexus underscores the challenge of achieving complete resection in multifocal schwannomas, where preservation of neurological function often necessitates subtotal excision.

Ongoing management focused on serial clinical and radiographic surveillance to monitor for interval progression, coupled with physical therapy to optimize strength and sensory recovery. Given the benign nature of most schwannomas and the patient’s stable neurological function, observation was favored over immediate re-exploration. Accordingly, the patient was placed on a structured surveillance protocol consisting of MRI with and without contrast at one to three months postoperatively, followed by imaging every three to four months during the first year, every three to six months for the subsequent year, and annually thereafter, coupled with clinical assessments at each interval [15,16]. Re-intervention will be considered if surveillance demonstrates radiographic progression, defined as >5-10% annual volumetric growth or >5 mm/year increase, or if the patient develops new or worsening pain, motor deficits, or sensory decline [17]. Although schwannomas are typically radioresistant, adjuvant options such as stereotactic body radiation therapy may be reserved for unresectable or progressively enlarging tumors, with targeted or systemic therapies employed only in rare, refractory cases [18].

This case underscores the importance of multidisciplinary collaboration in managing rare peripheral nerve sheath tumors. The combined expertise of neurosurgeons, radiologists, and pathologists was essential in diagnosing and safely resecting the tumor. Moreover, this case highlights the clinical value of advanced imaging in preoperative planning, helping to delineate tumor boundaries and avoid iatrogenic vascular or nerve injury.

Given the presence of four anatomically distinct schwannomas in this patient, the patient was referred to genetic medicine for formal testing for possible NF2, schwannomatosis-associated mutations (SMARCB1 and LZTR1), and NF1. At the time of this report, the results are still pending. A detailed family history was obtained and was noncontributory, with no known relatives affected by schwannomas, neurofibromatosis, or related tumor syndromes. The outcomes of genetic testing will guide long-term management, counseling, and surveillance strategies.

## Conclusions

This case highlights the unique challenges associated with the diagnosis and surgical management of multifocal brachial plexus schwannomas. Although schwannomas are generally benign and slow-growing, their occurrence within the brachial plexus presents significant anatomical and functional complexities. The patient’s postoperative course demonstrated that even with careful microsurgical dissection and intraoperative neuromonitoring, transient neurological deficits can occur due to manipulation of delicate nerve structures. However, the gradual recovery of strength and sensation underscores the capacity for functional improvement when neural architecture is preserved.

Persistent enhancement on postoperative imaging emphasizes the difficulty of achieving total resection in multifocal cases where the priority must remain preservation of neurological function. Accordingly, long-term management should include routine radiographic surveillance, ongoing functional assessment, and rehabilitation to promote optimal recovery. This case reinforces the value of a multidisciplinary approach, integrating neurosurgical, radiological, and pathological expertise, to ensure accurate diagnosis, safe intervention, and individualized postoperative care for patients with rare and complex peripheral nerve sheath tumors.
